# Prevalence of and factors associated with overweight and obesity in patients with severe mental disorders in Shenzhen: results from the urban Chinese population

**DOI:** 10.1017/S1368980024001988

**Published:** 2024-11-07

**Authors:** Wei Shan, Zhijian Zhou, Guojun Wang, Xiaodong Peng

**Affiliations:** 1 Affiliated Mental Health Center, Southern University of Science and Technology, Shenzhen, Guangdong, China; 2 Department of Public Health, Shenzhen Mental Health Center, Shenzhen Kangning Hospital, Shenzhen, Guangdong, China

**Keywords:** Severe mental disorders, Overweight, Obesity, China

## Abstract

**Objective:**

To determine the prevalence of overweight and obesity in patients with severe mental disorders (SMD) and the factors associated with their socio-demographic and disease characteristics in a cross-sectional population-based study.

**Design:**

This analysis examined the prevalence of overweight and obesity in 14 868 managed SMD patients in an urban area of Shenzhen city based on data from the health information monitoring system in 2021. Multivariate logistic regression were used to identify the factors associated with the prevalence of overweight and obesity in patients with SMD.

**Setting:**

China.

**Participants:**

14 868 patients with SMD.

**Results:**

The prevalence of overweight and obesity in patients with SMD in this study was 32·6 % and 16·1 %, respectively. In multivariate analysis, married status, Shenzhen household registration, management durations of 5–10 years and >10 years, participation in family physician services, taking clozapine or aripiprazole, FPG > 6·1 mmol/l, hypertension, TC ≥ 5·2 mmol/l, TG ≥ 1·7 mmol/l, and more frequent follow-ups in the past year were associated with higher odds of overweight and obesity. Compared to their respective reference categories, living with parents, spouse and children, taking risperidone, aripiprazole, amisulpride and perphenazine, FPG > 6·1 mmol/l, hypertension, TC ≥ 5·2 mmol/l, TG ≥ 1·7 mmol/l, and more frequent follow-ups in the past year were associated with higher odds of obesity.

**Conclusion:**

We observed a high prevalence of overweight and obesity in patients with SMD in this study. The findings highlight the need for integrated management of overweight and obesity risk factors among patients with SMD.

In China, severe mental disorders (SMD) are considered to be mental disorders with severe symptoms of mental illness that result in serious impairment of the patient’s social adaptation and other functions, incomplete recognition of his or her own health condition or objective reality, or inability to handle his or her own affairs, including schizophrenia, schizoaffective disorder, paranoid psychosis, bipolar disorder, epilepsy-induced mental disorder, and intellectual disability concomitant with psychotic disorder^(1)^. The burden of disease from SMD continues to rise and is associated with poorer health outcomes and increased mortality, in addition to contributing to most of the prevalence of mental health disability^(2)^. The average death rate of patients with SMD is 2–3 times higher than that of the general population, and their life expectancy is shortened by 10–20 years^(3)^. Although people with SMD are at higher risk of death from unnatural causes than the general population, the majority of deaths among people with SMD are due to physical health conditions, both noncommunicable and communicable^(4)^. In addition, patients with SMD are more likely to have lifestyles that include risk factors for noncommunicable diseases, such as lack of physical activity and unhealthy diet^(5)^.

Obesity and overweight are major risk factors for chronic noncommunicable diseases. In 2019, the number of deaths due to chronic noncommunicable diseases in China accounted for 88·5 % of the total deaths, and the base number of patients with chronic diseases is still expanding^(6,7)^. Obesity is considered an independent risk factor for CVDs. Not only does it greatly increase the risk of CVDs such as hypertension, CHD, myocardial infarction, angina pectoris and stroke, but it is also associated with increased mortality from CVDs^(8)^. Patients with SMD are more severely affected by overweight and obesity, and the increased rate of obesity is higher than that of the general population^(9)^. A survey in European and American countries reported that 26–55 % of SMD patients were obese, and the obesity rate of schizophrenia patients was approximately 4·3 times that of the general population^(10,11)^. A recent cross-sectional study in China reported that the obesity rate of schizophrenia was 16·4–20·9 %^(12,13)^. which was higher than that of the general population^(14)^. There is growing evidence that obesity and overweight lead to an increased incidence of CVD, which in turn is the most common cause of SMD premature natural death^(15)^. Therefore, obesity and overweight pose a greater potential threat to the physical health of patients with SMD.

However, SMD for a variety of reasons has led to high obesity and overweight rates. Growing evidence has reported an association between the use of second-generation antipsychotics (SGAs) and an increased risk of obesity, weight gain, type 2 diabetes, and increased risk of dyslipidemia^(16)^. Lack of exercise^(17)^, a sedentary lifestyle^(18)^, poor diet^(19)^, and adverse life events in childhood are also important factors affecting obesity and overweight in patients with SMD^(20)^. Other important factors include the genetic susceptibility of patients and several psychosocial and socio-economic risk factors, such as being female^(21)^, older and having a lower educational level^(22)^. In recent decades, despite a series of governmental policies to improve the health status of patients with SMD living in the community, the overall quality of life remains relatively low^(23)^.

Most studies have focused on schizophrenia, but there are relatively few reports on bipolar disorder, schizoaffective disorder, paranoid psychosis, epileptic-induced mental disorder, and intellectual disabilities that are associated with mental disorders^(24)^. In China, few large-sample studies have been reported on the physical health of community-dwelling patients with SMD who were managed by a health monitoring information system. Considering that patients with SMD return to the community after hospital discharge or outpatient visits, studying the physical health of such populations will be of great public health significance for targeted interventions and adjustment of health management strategies. The purpose of the present study was to examine the prevalence of overweight and obesity in patients with SMD and to identify the association between overweight and obesity and socio-demographic and clinical factors in these patients.

## Methods

### Data sources

All data for this study were obtained from the *Shenzhen Mental Health Prevention and Control Management System*. Shenzhen is a coastal city located in the southern part of China’s Guangdong Province, with one of the country’s leading economies, a resident population of 17,681,600 (13·9 % of Guangdong’s population), and a highly mobile population. Based on China’s household registration system, the ‘Hukou’ policy, non-Shenzhen household registration or the ‘floating population’ are defined as people who leave their registered residence areas (e.g. cities, towns and villages) to engage in various jobs in non-residence areas. Coastal urban cities, such as Shenzhen in the Pearl River Delta area, are the major destinations of internal migration. By the end of December 2021, a total of 32 349 patients with severe mental disorders were registered in the *Shenzhen Mental Health Prevention and Control Management System*. There were 31 580 patients (97·62 %) who accepted the community health management follow-up service, 448 patients (1·38 %) refused services, and 321 patients (0·99 %) missed visits. This information monitoring system was established and started to operate in 2006. The data system is managed by the professional staff of Shenzhen Mental Health Center.

First, patients with SMD were screened and reported in two ways. One involved the screening and assessment of suspected patients by community workers based on the Abnormal Behavior Inventory and inviting psychiatrists to confirm the diagnosis^(1)^. The other was an assessment and diagnosis by psychiatrists in medical institutions (psychiatric and nonpsychiatric hospitals) based on the International Statistical Classification of Diseases and Related Health Problems (10th edition) (ICD-10)^(25)^. When a diagnosis of SMD (i.e. schizophrenia, bipolar disorder, schizoaffective disorder, paranoid psychosis, epilepsy-induced mental disorder, and intellectual disability associated with mental disorder) was confirmed, the community health management program for SMD was explained^(26)^. After the patient and guardian fully understood the program, they voluntarily participated and signed an informed consent form, and then the psychiatrist registered the report in the *Shenzhen Mental Health Prevention and Control Management System*, and the relevant information was transferred to the primary mental health institution in the area where the patient lived.

Second, health management cases for patients diagnosed with SMD were established. Patients were contacted by general practitioners from primary mental health institutions after uniform training and assessment to inform them about community health management and mental health policies. Standardised tools were used to collect socio-demographic information and assess the patient’s mental status^(1)^. After the individual health management case was established, the patient was formally integrated into the community health management project.

Third, follow-up was conducted. General practitioners in primary mental health institutions start the first follow-up assessment of patients when they establish community health records. The follow-up assessment includes the patient’s psychiatric symptoms, violence risk, insight, medication compliance, adverse drug reactions, social functioning and physical health status. After each follow-up assessment of the patients by the general practitioners in primary mental health institutions, targeted health interventions are carried out according to the conditions and needs of the patients, such as medication adjustment, management of medication side effects, guidance on psychiatric rehabilitation training, psychological counselling, health education and care skills for family members, guidance on healthy lifestyles, publicising the government’s mental health support policy, etc. The frequency of follow-up depends on the patient’s disease condition. The criteria for disease stable are the absence of obvious psychiatric symptoms, no risk of violence, no adverse drug reactions or serious physical illness, good insight and social functioning. The criterion for a basically stable condition is the presence of risky behaviour or psychiatric symptoms, with at least one aspect of insight or social functioning being poor. The criteria for instability are the presence of overtly risky behaviour or psychiatric symptoms, lack of insight, serious medication side effects, or serious physical illness. During the follow-up period, patients could apply to enter or leave the cohort at any time. The study was approved by the Medical Ethics Review Committee of Shenzhen Kangning Hospital.

### Study measures

We used BMI as the main outcome measure in the study of physical health management of SMD. Patients with SMD who were registered in the Shenzhen Mental Health Prevention and Control Management System not only received regular mental health assessments and health advice but also received an annual health check-up if desired, all of which were free of charge^(27)^. When a patient makes an appointment for a physical exam at a primary mental health facility, the general practitioner measures height and weight, and the patient wears light clothing and no shoes. BMI was defined as the weight (kg) divided by the square of height (m^2^). Overweight and obesity are defined according to the cutoff values of BMI within the Chinese population^(28)^. A BMI of <23·9 kg/m^2^ is considered normal weight or underweight while BMI ≥ 24–27·9 kg/m^2^ is considered overweight and BMI ≥ 28 kg/m^2^ is considered obese.

### Statistical analysis

Statistical analyses were performed using SPSS version 21·0 and R version 3·5·1. We used the mean or median, as appropriate, to summarise the measured variables, while the count variables are reported as frequencies and percentages. The normality of continuous variables was tested with the Shapiro‒Wilk test. The measurement data were analysed using an independent *t* test and Wilcoxon rank test according to the normal distribution test, and the chi-square test was used for count data. One-way binary logistic regression was used to assess OR and 95 % confidence intervals for factors associated with overweight (BMI ≥ 24 kg/m^2^ *v*. BMI < 24 kg/m^2^) and obesity (BMI ≥ 28 kg/m^2^ *v*. BMI < 28 kg/m^2^). Variables that were statistically significant in the univariate analysis were again entered into a multivariate logistic regression model. Forest plots were generated using the R package ‘forest plot’. A two-sided α = 0·05 was used as the test level, and *P* < 0·05 was considered to indicate statistical significance.

## Results

### General characteristics of the study population

The study included 14 868 patients with SMD: 6932 males and 7936 females (Table 1). The mean values of age, duration of untreated psychosis (DUP), and number of years in the register were 41·79 years, 0·39 years, and 5·44 years, respectively; 8126 patients (54·7 %) were married, 7356 (49·5 %) were unemployed, 3340 (22·5 %) were in primary school and below, 1193 (8·0 %) were living alone, 8881 (8·0 %) were nonlocal household members (59·7 %), 9388 (63·1 %) were without medical insurance, and 12 006 (80·8 %) had a nonpoor family economy; the disease diagnosis was most often schizophrenia (57·2 %), followed by bipolar disorder (24·7 %); and 12 037 (81·0 %) participated in family physician services. There were 4841 (32·6 %) patients with overweight and 2396 (16·1 %) patients with obesity; the mean values of fasting blood glucose, TC and TG were 5·30 mmol/l, 4·74 mmol/l and 1·44 mmol/l, respectively. The mean values of fasting glucose, TC, and TG were 5·30 mmol/l, 4·74 mmol/l, and 1·44 mmol/l, respectively. SBP and DBP were 118·59 mmHg and 75·27 mmHg, respectively (Table 1). There were significant differences between men and women in age, years of enrollment management, marriage, employment status, level of education, living status, disease diagnosis, participation in family physician services, fasting glucose, TC, TG, BMI, SBP, and DBP.

**Table 1:** Sociodemographic and physiological characteristics of the study population from 2006 to 2021

Variables	All (%), *n* 14868		Male (%), *n* 6932		Female (%), *n* 7936		Statistics	*P* value
	Mean or median	sd or interquartile range	Mean or median	sd or interquartile range	Mean or median	sd or interquartile range		
Age^*^ (year)	41·79	14·17	39·32	13·51	43·95	14·39	–20·238	0·000
DUP^†^ (year)	0·39	0·08, 3·05	0·37	0·08, 3·07	0·41	0·08, 3·04	–0·866	0·387
Number of years in the register^*^	5·44	3·31	5·56	3·38	5·33	3·24	4·301	0·000
FPG^†^ (mmol/l)	5·30	4·82, 5·86	5·27	4·80, 5·80	5·30	4·89, 5·90	–5·147	0·000
TC^†^ (mmol/l)	4·74	4·09, 5·46	4·72	4·05, 5·44	4·75	4·12, 5·48	–2·278	0·023
TG^†^ (mmol/l)	1·44	0·98, 2·18	1·56	1·04, 2·38	1·34	0·92, 2·02	–12·178	0·000
BMI (kg/m^2^)	24·32	4·07	24·35	3·89	24·30	4·22	0·813	0·416
BMI							17·062	0·000
Normal	7631	51·3	3479	50·2	4152	52·3		
Overweight	4841	32·6	2374	34·2	2467	31·1		
Obesity	2396	16·1	1079	15·6	1317	16·6		
SBP^*^ (mmHg)	118·59	13·98	120·26	12·92	117·13	14·69	13·815	0·000
DBP^*^ (mmHg)	75·27	9·90	76·42	9·90	74·26	9·80	13·346	0·000
Marriage							585·339	0·000
Single/divorced/widowed	6742	45·3	3876	55·9	2866	36·1		
Married	8126	54·7	3056	44·1	5070	63·9		
Employment							111·847	0·000
Unemployed	7356	49·5	3108	44·8	4248	53·5		
Employed	7512	50·5	3824	55·2	3688	46·5		
Education							95·195	0·000
Primary school and below	3340	22·5	1312	18·9	2028	25·6		
Junior high school	5303	35·7	2554	36·8	2749	34·6		
High school	3722	25·0	1847	26·6	1875	23·6		
College school and above	2503	16·8	1219	17·6	1284	16·2		
Living status							155·794	0·000
Alone	1193	8·0	651	9·4	542	6·8		
With parents, spouse and children	12 137	81·6	5374	77·5	6763	85·2		
With siblings and relatives	1084	7·3	623	9·0	461	5·8		
With friends and fellow villagers	412	2·8	261	3·8	151	1·9		
With others	42	0·3	23	0·3	19	0·2		
Type of household registration							2·445	0·118
Local	5987	40·3	2838	40·9	3149	39·7		
Nonlocal	8881	59·7	4094	59·1	4787	60·3		
Medical insurance							2·251	0·134
No	9388	63·1	4333	62·5	5055	63·7		
Yes	5480	36·9	2599	37·5	2881	36·3		
Family financial status							0·023	0·880
Below the local poverty standard	2862	19·2	1338	19·3	1524	19·2		
Nonpoverty	12 006	80·8	5594	80·7	6412	80·8		
Disease diagnosis							221·205	0·000
Schizophrenia	8507	57·2	3695	53·3	4812	60·6		
Bipolar disorder	3675	24·7	1760	25·4	1915	24·1		
Schizoaffective disorder	666	4·5	266	3·8	400	5·0		
Delusional disorder	146	1·0	58	0·8	88	1·1		
Psychotic disorder due to epilepsy	421	2·8	246	3·5	175	2·2		
Intellectual disability with mental disorders	1453	9·8	907	13·1	546	6·9		
Participate in family physician services							4·368	0·037
No	2831	19·0	1270	18·3	1561	19·7		
Yes	12 037	81·0	5662	81·7	6375	80·3		

* Mean (sd).

† Median (interquartile range).

DUP, duration of untreated psychosis; FPG, fasting plasma glucose; TC, total cholesterol; TG, triglyceride; SBP, systolic blood pressure; DBP, diastolic blood pressure.

### Logistic regression analysis of overweight and obesity

Table 2 shows the univariate logistic regression analysis for obesity or overweight. We found that age, marriage, education level, living status, type of household registration, schizophrenia, intellectual disability concomitant with psychotic disorder, DUP, years of enrollment management, participation in family physician services, olanzapine, clozapine, risperidone, aripiprazole, amisulpride, FPG, hypertension, TC, TGs, and number of follow-ups in the past year were associated with overweight and obesity. Therefore, we included these factors in a multivariate logistic regression model for analysis.

**Table 2:** Results of univariate logistic regression analysis for BMI

variables	Overweight and obesity				Obesity			
	Number of patients (*n* 7237)	OR	95 % CI	*P* value	Number of patients (*n* 2396)	OR	95 % CI	*P* value
Sex (Ref: Male)	3784	0·92	0·86, 0·98	0·010	1317	1·08	0·99, 1·18	0·088
Age (Ref: 18–44)								
45–59	2335	1·41	1·31, 1·51	0·000	729	1·07	0·97, 1·18	0·195
≥60	899	1·45	1·12, 1·38	0·000	262	0·92	0·80, 1·06	0·240
Marriage (Ref: Single/divorced/widowed)	4273	1·41	1·33, 1·51	0·000	1372	1·13	1·04, 1·24	0·005
Employment (Ref: Unemployed)	3658	1·00	0·94, 1·07	0·960	1143	0·87	0·80, 0·95	0·003
Education (Ref: Primary school and below)								
Junior high school	2720	1·06	0·97, 1·15	0·211	888	0·90	0·81, 1·01	0·081
High school	1755	0·90	0·82, 0·98	0·021	582	0·83	0·74, 0·94	0·004
College and above	1095	0·78	0·70, 0·87	0·000	318	0·65	0·57, 0·76	0·000
Living status (Ref: Alone)								
With parents, spouse and children	6069	1·35	1·20, 1·52	0·000	2041	1·47	1·23, 1·76	0·000
With siblings and relatives	497	1·14	0·97, 1·35	0·117	162	1·28	1·01, 1·63	0·045
With friends and fellow villagers	145	0·73	0·58, 0·92	0·009	47	0·94	0·66, 1·33	0·720
With others	18	1·01	0·54, 1·88	0·972	2	0·36	0·09, 1·52	0·167
Type of household registration (Ref: Nonlocal)	4443	1·14	1·07, 1·22	0·000	1466	1·08	0·98, 1·18	0·113
Medical insurance (Ref: No)	2621	0·95	0·89, 1·01	0·115	840	0·91	0·83, 1·00	0·046
Family financial status (Ref: Below the local poverty standard)	5788	0·91	0·84, 0·99	0·020	1885	0·86	0·77, 0·95	0·005
Disease diagnosis								
Schizophrenia (Ref: No)	4284	1·17	1·10, 1·25	0·000	1417	1·10	1·01, 1·20	0·038
Bipolar disorder (Ref: No)	1777	0·98	0·91, 1·06	0·653	550	0·89	0·80, 0·99	0·029
Schizoaffective disorder (Ref: No)	348	1·16	0·99, 1·36	0·059	123	1·19	0·97, 1·45	0·091
Delusional disorder (Ref: No)	65	0·85	0·61, 1·17	0·313	17	0·68	0·41, 1·14	0·142
Psychotic disorder due to epilepsy (Ref: No)	186	0·83	0·68, 1·01	0·062	63	0·91	0·70, 1·20	0·515
Intellectual disability with mental disorders (Ref: No)	577	0·67	0·60, 0·75	0·000	226	0·95	0·82, 1·11	0·540
DUP (Ref: <2 year)								
2–5 year	763	1·01	0·91, 1·13	0·862	252	1·00	0·87, 1·16	0·999
5–10 year	493	0·92	0·81, 1·05	0·196	171	1·01	0·84, 1·19	0·993
>10 year	751	1·20	1·07, 1·34	0·002	234	1·03	0·89, 1·20	0·711
Number of years in the register (Ref:<2 year)								
2–5 year	1975	1·16	1·05, 1·29	0·003	649	1·11	0·96, 1·27	0·150
5–10 year	3433	1·24	1·13, 1·36	0·000	1137	1·16	1·02, 1·32	0·021
>10 year	666	1·40	1·22, 1·60	0·000	228	1·30	1·08, 1·56	0·005
Participate in family physician services (Ref: No)	5974	1·22	1·13, 1·33	0·000	2001	1·23	1·09, 1·38	0·001
Olanzapine (Ref: No)	2128	1·09	1·01, 1·17	0·019	673	0·98	0·89, 1·08	0·617
Clozapine (Ref: No)	1064	1·41	1·28, 1·56	0·000	352	1·22	1·08, 1·38	0·002
Risperidone (Ref: No)	1545	1·21	1·11, 1·31	0·000	543	1·23	1·10, 1·36	0·000
Quetiapine (Ref: No)	808	1·00	0·90, 1·11	0·980	274	1·03	0·90, 1·19	0·638
Ziprasidone (Ref: No)	126	1·10	0·86, 1·41	0·459	50	1·33	0·97, 1·82	0·076
Aripiprazole (Ref: No)	885	1·11	1·00, 1·22	0·049	323	1·22	1·07, 1·39	0·003
Amisulpride (Ref: No)	356	1·23	1·06, 1·44	0·008	135	1·35	1·11, 1·64	0·002
Perphenazine (Ref: No)	200	1·47	1·18, 1·82	0·001	81	1·62	1·26, 2·08	0·000
Sulpiride (Ref: No)	217	1·20	0·98, 1·46	0·073	67	1·02	0·78, 1·33	0·882
Lithium carbonate (Ref: No)	635	1·10	0·98, 1·23	0·116	213	1·08	0·92, 1·26	0·353
FPG (Ref: ≤6·0 mmol/l)	1844	2·20	2·02, 2·39	0·000	723	2·08	1·88, 2·30	0·000
Hypertension (Ref: No)	857	2·45	2·17, 2·78	0·000	376	2·46	2·16, 2·80	0·000
TC (Ref: < 5·1 mmol/l)	2057	1·76	1·62, 1·91	0·000	745	1·60	1·44, 1·77	0·000
TG (Ref: < 1·6 mmol/l)	2704	2·92	2·69, 3·17	0·000	1025	2·49	2·25, 2·77	0·000
Number of follow-ups in the past year (Ref: ≤4)								
5–8	3640	1·37	1·22, 1·53	0·000	1133	1·44	1·21, 1·71	0·000
9–12	2490	1·48	1·29, 1·70	0·000	856	1·70	1·40, 2·08	0·000
≥13	937	1·71	1·40, 2·10	0·000	353	2·03	1·55, 2·65	0·000
Number of face-to-face follow-ups in the past year (Ref: ≤2)								
3–4	365	0·99	0·67, 1·46	0·957	115	1·08	0·60, 1·96	0·789
5–6	2209	1·31	0·91, 1·89	0·152	671	1·25	0·71, 2·19	0·438
≥7	4615	1·58	1·10, 2·29	0·014	1596	1·64	0·94, 2·87	0·084

DUP, duration of untreated psychosis; FPG, fasting plasma glucose; TC, total cholesterol; TG, triglyceride.

**Fig. 1:**
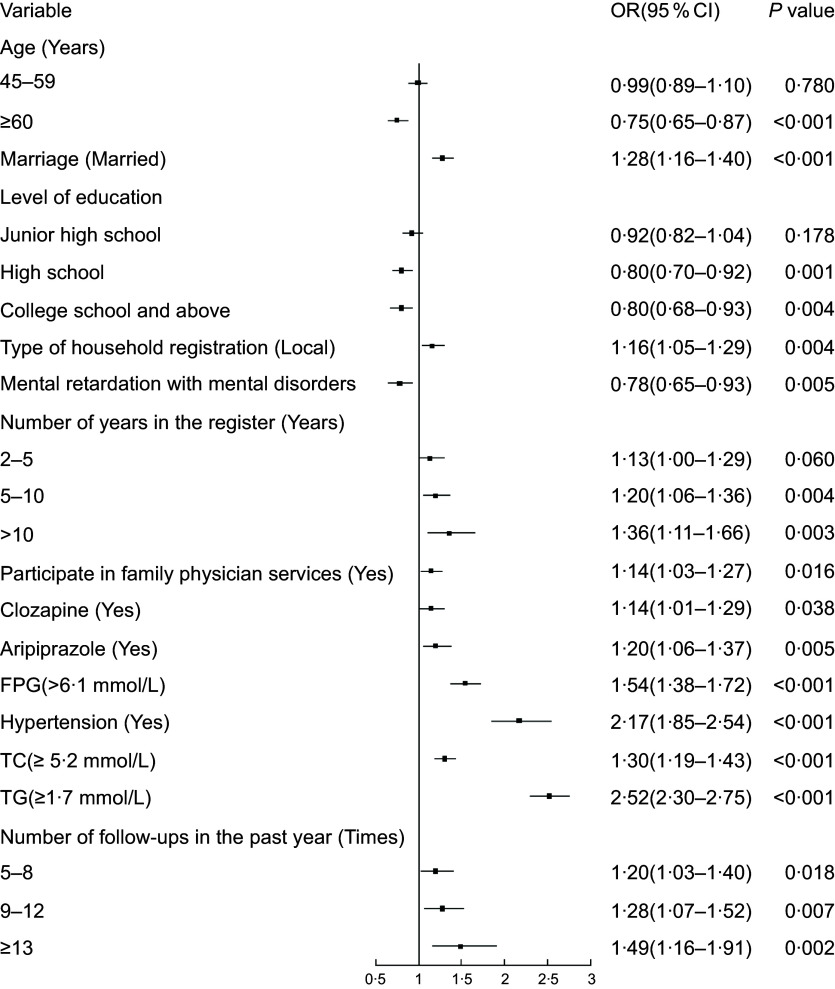
Results of multiple logistic regression analysis for overweight and obesity. FPG, fasting plasma glucose; TC, serum total cholesterol; TG, triglyceride

Multivariate logistic regression analysis showed (Fig. 1) that individuals aged ≥ 60 years, with education levels of high school and college and above, and intellectual disability concomitant with mental disorders had lower odds of being overweight and obese. Being married, Shenzhen household registration, number of years of management 5–10 years, number of years of management >10 years, participation in family physician services, clozapine, aripiprazole, FPG, hypertension, TC, TGs, and number of follow-ups in the past year were associated with higher odds of overweight and obesity.

### Logistic regression analysis of obesity

Table 2 shows the univariate logistic regression analysis for obesity. We found that marriage, employment status, level of education, living status, medical insurance, family financial status, schizophrenia, bipolar disorder, number of years in the register, participation in family physician services, clozapine, risperidone, aripiprazole, amisulpride, perphenazine, FPG, hypertension, TC, TGs, and number of follow-ups in the past year were associated with obesity. Therefore, we included these factors in a multivariate logistic regression model for analysis.

**Fig. 2:**
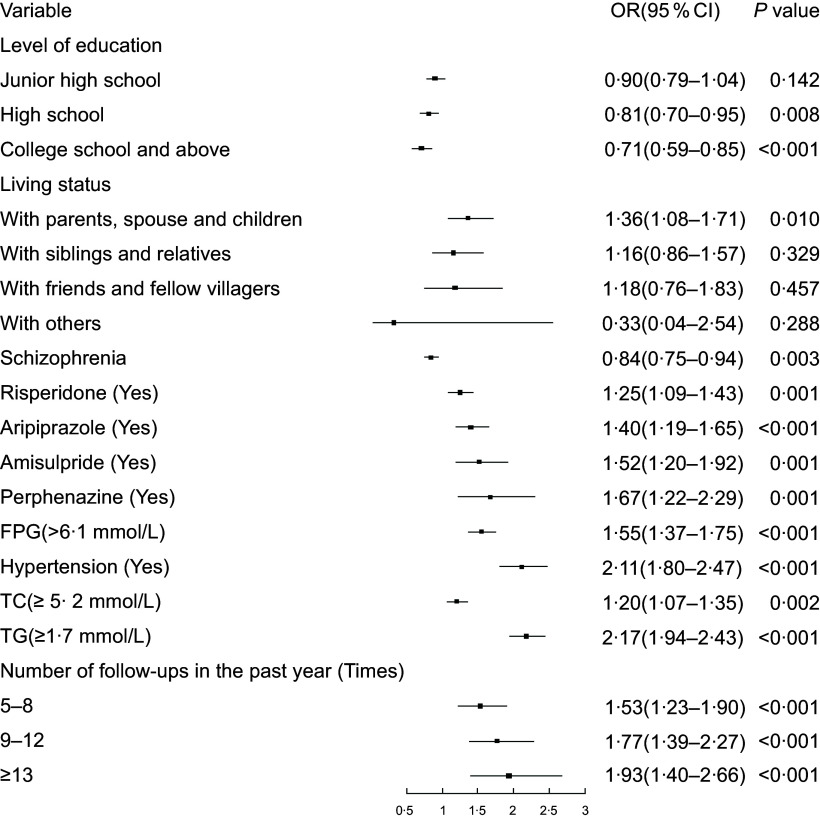
Results of multiple logistic regression analysis for obesity. FPG, fasting plasma glucose; TC, serum total cholesterol; TG, triglyceride

Multivariate logistic regression analysis showed that education levels of high school, college, and above and schizophrenia were associated with lower odds of obesity. Living status with parents, spouse and children, risperidone, aripiprazole, amisulpride, perphenazine, FPG, hypertension, TC, TGs, and number of follow-ups in the past year were associated with higher odds of obesity (Fig. 2).

## Discussion

This is the first community-based study to explore the physical health of patients with SMD in China. The results of the study provide important scientific guidance for improving community management strategies and measures for SMD and improving the physical health of patients. In this study, we found that approximately half of the participants were overweight and obese. Married status, Shenzhen household registration, management of 5–10 years, management of > 10 years, participation in family physician services, clozapine, aripiprazole, FPG, hypertension, TC, TG, and number of follow-ups in the past year were associated with a higher risk of overweight and obesity. Age ≥ 60, high school educational status, college and above educational status, and intellectual disability concomitant with mental disorders were associated with a lower likelihood of being overweight or obese. All these data suggest that obesity in patients with SMD has become a serious public health burden for the Chinese population and needs more attention and intervention.

We found that the prevalence of obesity in patients with SMD in Shenzhen was close to the prevalence of obesity in the general population but higher than the average of the general population in large cities^(29)^. Consistent with our findings, a meta-analysis concluded that people with severe mental illness had higher odds of obesity than the general population in all regions^(30)^. These findings are also similar to those of an obesity survey of schizophrenia patients in China (16 %–20 %)^(12,31)^. Meanwhile, we found that the prevalence of overweight in patients with SMD in Shenzhen was lower than that of the general population and that of the general population in large cities but was close to the global findings on the overweight rate of patients with SMD^(29,30)^. A study from 39 034 rural adults showed that the overweight rate in the rural population was 34·5 % and the obesity rate was 16·8 %, both of which were higher than those observed in our study^(32)^. The possible reasons for this phenomenon are as follows. First, our research population is concentrated in Shenzhen, a city with a 100 % urbanisation rate in China, and its economy is relatively developed. Residents have more labor opportunities and exercise space, so they can exercise more. Second, urban residents have access to more medical resources and more health knowledge. Since 2010, the Chinese government has carried out a number of national prevention and control programs for obesity^(33)^. The results showed that interventions tend to be effectively implemented in urban areas but that they have little effect in rural areas^(34)^. Third, population migration leads to a change in diet structure, and Shenzhen is dominated by an internal migrant population. Related studies have shown^(35)^ that changes in diet structure are more likely to lead to obesity, especially changes toward animal-based diets, and urban residents are more likely to take in more protein. Fourth, patients with SMD have a sense of stigma^(36)^, and the stress caused by low income, unemployment and discrimination can not only affect their recovery and life but can also result in obesity^(37)^.

Although antipsychotic drugs can better treat psychiatric disorders, they can also cause a number of problems, such as metabolic disorders^(38)^. In our study, patients with SMD treated with clozapine and aripiprazole were more likely to be overweight and obese, while patients with SMD treated with perphenazine, risperidone, aripiprazole and amisulpride were more likely to have obesity. This finding is consistent with most research reports that antipsychotics might reduce caloric utilisation in the body, leading to an increased appetite in patients who, in turn, lack sufficient exercise, leading to weight gain^(38,39)^. Previous studies^(8,40)^ have reported that obesity and overweight can lead to vascular aging, dyslipidemia and carotid atherosclerosis, impaired glucose tolerance, and abnormal blood glucose, increasing the risk of hypertension^(41)^, coronary atherosclerotic heart disease^(42)^, type 2 diabetes^(43)^, stroke and many different cancers^(44)^. In this study, we found an association between overweight and obesity and hypertension, TC, TG^(31)^, and FPG^(8)^ in patients with SMD, suggesting an increased risk of CVD.

Interestingly, not all patients with SMD are prone to obesity and overweight. Our study found that intellectual disability concomitant with mental disorders was associated with a lower risk of obesity and overweight, while schizophrenia was associated with a lower risk of obesity. Most patients with intellectual disabilities have difficulty in self-care and require care from others, and those with severe disabilities can become completely disabled, with refusal to eat and occasional self-vomiting likely to be the main causes of this phenomenon^(45)^, which is more likely to occur in patients with intellectual disabilities concomitant with mental disorders. More than half of the patients with SMD participating in this study were community-dwelling patients with schizophrenia, and most of them were nonlocal residents who needed to participate in work to pay for various living expenses and medical expenses^(46)^.

However, physical health management in community mental health services needs more attention in the future. Our study found that patients with SMD who participated in family physician services, had longer years of management, and had more follow-up visits were more likely to be overweight and obese. The possible reason is that community mental health services in China have always been focused on providing better mental health services and rehabilitation services for patients with SMD while paying less attention to their physical health and lack of relevant physical health management^(47)^. At present, family physician services are in the initial stage in China, and there are still many deficiencies. Meanwhile, there is a large gap in the quality assurance of primary health care in China, and the education and training of primary health care practitioners are poor, which requires a more complete employee training system and higher training quality^(48)^.

In addition, our study identified several risk factors for obesity in Chinese patients with SMD. Married patients with SMD were more likely to have obesity and overweight. Studies have shown that having a partner is more likely to lead to overweight and obesity^(49)^. Men and women are more than twice as likely to become obese after marriage compared with single people, and obesity in one spouse will increase the risk of obesity in the other. At the same time, we also found that patients with Shenzhen household registration who lived with parents, spouses, or children were more prone to overweight and obesity. The possible reason for this phenomenon is that patients with registered residence in Shenzhen have better treatment in social welfare, medical insurance, housing and other aspects than those without registered residence^(28)^. Patients living with parents, spouses or children may receive better care and easily form a sedentary and eating lifestyle, which leads to obesity and overweight. Furthermore, in our study, patients with SMD with higher education were less likely to be overweight and obese than those with lower education. Previous studies have confirmed that people with low education are more likely to be obese and overweight^(50)^. People’s education will affect their way of life. People with higher levels of education may pay more attention to maintaining physical fitness and a healthy diet and are more willing to leave the home.

The advantage of our study is that we provided population-based data on the physical health of patients with SMD in Chinese communities. However, there are still several limitations in this study. First, this study has a cross-sectional design, and the patients with severe mental disorders involved in this study were all receiving community mental health services, so more epidemiological investigations with large samples are needed to obtain more representative research results. Second, there are no variable data in the study, such as exercise habits, eating habits, lifestyle habits, genomics data, use of other antipsychotics and other diseases and their drug treatments (such as CVD, diabetes, glucose-lowering drugs, and antihypertensive drugs), which are considered to be potential risk factors for an increased BMI.

## Conclusions

In summary, we reported a high prevalence of overweight and obesity in patients with SMD in the community, and their physical health was shown to be often neglected. Our study provides insights into interventions for controllable risk factors for overweight and obesity in Chinese patients with SMD. Research on the physical health of this population will have great public health significance for targeted interventions and adjustment of health management strategies, and more efforts should be made to improve the overall management of people with SMD in urban communities.
